# Association between neutrophil to lymphocyte ratio and the mortality of patients with sepsis: an update systematic review and meta-analysis

**DOI:** 10.3389/fmed.2025.1637365

**Published:** 2025-10-20

**Authors:** Zimo Gao, Xiaolei Wang, Wenfeng Wang, Zhichen Kang, Xinghai Chen

**Affiliations:** ^1^Department of Emergency Medicine‌, The Second Hospital of Jilin University, Jilin, China; ^2^Department of ‌Intensive Medicine‌, The Second Hospital of Jilin University, Jilin, China; ^3^Department of ‌Rehabilitation, The Second Hospital of Jilin University, Jilin, China

**Keywords:** neutrophil to lymphocyte ratio, sepsis, mortality, systematic review, meta-analysis

## Abstract

**Objectives:**

To present the most up-to-date systematic review and meta-analysis assessing the relationship between the neutrophil-to-lymphocyte ratio (NLR) and mortality in individuals diagnosed with sepsis.

**Methods:**

We conducted a systematic literature retrieval via PubMed, Embase, Web of Science, and Cochrane Library until March, 2025 for studies which evaluated the link between NLR and the mortality of patients with sepsis. Odds ratios (OR) and 95% confidence intervals (CI) were used for data pooling. In addition, sensitivity analysis and subgroup analysis were performed to examine the stability of the results and potential sources of heterogeneity. All analyses were performed using Review Manager 5.4 and STATA 15.1.

**Results:**

A total of 21 studies including 23,621 patients were incorporated into the meta-analysis. The results demonstrated a significantly higher mortality (OR: 1.11; 95% CI: 1.08, 1.14; *p*<0.00001) in the group with high NLR compared with the group with low NLR. The sensitivity analysis confirmed the stability of this result. In addition, subgroup analysis by cut-off and sample size showed that the predictive value of NLR for mortality was still significant in all subgroups (*p* < 0.05).

**Conclusion:**

NLR was significantly associated with the risk of death in individuals diagnosed with sepsis. The higher the NLR, the higher the risk of death. Considering the potential publication bias and inevitable heterogeneity of this study, further large-sample, multicenter, prospective clinical studies are needed in the future to verify the exact link between NLR and the risk of death in patients with sepsis.

**Systematic review registration:**

Our systematic review has been registered in the International Prospective Register of Systematic Reviews (PROSPERO). The unique identifier is CRD420251050651, and the publicly accessible URL is https://www.crd.york.ac.uk/PROSPERO/view/CRD420251050651.

## 1 Introduction

Sepsis is defined as a life-threatening organ dysfunction caused by a dysregulated host response to infection (1). Genes encoding inflammatory cytokines, signal transduction factors, and cell adhesion molecules are overactivated, causing a dramatic increase in inflammatory cytokines, manifested as immune hyperfunction, resulting in one or more organ dysfunction, which is the main cause of early death in individuals diagnosed with sepsis (2). Every year, around 31.5 million people across the globe are affected by sepsis, leading to approximately 5.3 million deaths (3). In the United States, an estimated 1 million new sepsis cases are reported each year (4). in regions such as Western Asia, sepsis is diagnosed in over 41.76% of ICU admissions, with a mortality rate exceeding 55.8% (5). The death rate from sepsis tends to be even higher in Asian countries like China and India compared to European nations (6). In recent years, with the implementation of sepsis management guidelines and the improvement of systematic and procedural monitoring, diagnosis, and management of sepsis, these measures have contributed to a significant reduction in early mortality rate of individuals diagnosed with sepsis (7). Unfortunately, the long-term outcomes of sepsis survivors have not improved over time. About 50% of individuals diagnosed with sepsis recover, one-third die within a year, and one-sixth suffer severe, lasting damage (8). Therefore, it is important to assess the stage of sepsis in patients early and understand the pathophysiology of the disease.

The neutrophil to lymphocyte ratio (NLR) reflects the status of the innate and adaptive immune systems. Neutrophils, as the core of innate immunity, clear pathogens through phagocytosis and release inflammatory mediators; while lymphocytes, as the key to adaptive immunity, coordinate specific immune responses, produce antibodies and perform immune regulation by differentiating into different subsets (such as T cells and B cells). Therefore, NLR may serve as an indicator to measure the balance between innate immune activation and adaptive immune suppression (9). Therefore, the NLR may serve as an indicator of the interplay between innate and adaptive immune responses. As a novel marker of inflammation, NLR is a reliable parameter for describing immune responses to various stimuli (10, 11). NLR is calculated by dividing the absolute neutrophil count by the absolute lymphocyte count (12), which is an easy-to-use and efficient parameter. In recent years, the value of NLR in the diagnosis and prognosis of inflammatory diseases has attracted much attention (13, 14). An elevated NLR can effectively reflect the body’s inflammatory and stress response state, and demonstrates high sensitivity in detecting systemic inflammation (15).

The meta-analysis by Wu et al. (16) analyzed studies published before 2023, and the results showed that NLR is a reliable and valuable biomarker for prediction of the prognosis and mortality risk of adults with sepsis. However, since the publication of Wu’s study (16), several large-scale clinical studies investigated the prognostic importance of NLR in individuals with sepsis, and the conclusions were inconsistent. Therefore, this study re-evaluates the prognostic value of NLR for mortality in individuals with sepsis through a systematic literature review and meta-analysis. The goal is to provide a solid evidence base for improving the risk stratification of patients with sepsis and formulating individualized intervention strategies, and to inform future translational applications of inflammatory markers in sepsis management.

## 2 Methods

### 2.1 Literature search

Our meta-analysis was performed in accordance with the 2020 PRISMA and registered in the PROSPERO (CRD420251050651). The search was conducted via PubMed, Embase, Web of Science, Cochrane Library up to March 2025 for studies that focused on the association between NLR and the mortality in individuals with sepsis. To identify relevant studies, we employed a comprehensive search strategy with the following keywords: “Neutrophils,” “Lymphocytes,” “Mortality,” “Death” and “Sepsis.” The PubMed search methodology is outlined below: (((((“Neutrophils”[Mesh]) OR (((Neutrophil) OR (Polymorphonuclear Leukocyte)) OR (LE Cell))) AND ((“Lymphocytes”[Mesh]) OR ((Lymphocyte) OR (Lymphoid Cell)))) AND (Ratio)) AND ((“Sepsis”[Mesh]) OR ((((((Bloodstream Infection) OR (Septicemia)) OR (Blood Poisoning)) OR (Pyemia)) OR (Pyaemia)) OR (Pyohemia)))) AND ((“Mortality”[Mesh]) OR (((Mortalities) OR (Death Rate)) OR (Death))). As part of our comprehensive search process, we also manually examined the bibliographies of all eligible studies. The selection of relevant literature was conducted independently by two reviewers, and any inconsistencies were resolved through discussion. Details of the complete search protocol can be found in Supplementary Table S1.

### 2.2 Inclusion and exclusion criteria

The studies included in the analysis fulfilled the following: (1) the research design had to be a cohort study, case–control study, or randomized controlled trial; (2) the study population consisted of patients diagnosed with sepsis; (3) the primary objective was to determine the association between NLR and sepsis-related mortality; and (4) sufficient multivariate data were available to calculate odds ratios (ORs) with corresponding 95% confidence intervals (CIs). Manuscripts categorized as study protocols, unpublished works, non-original publications (such as letters, editorials, abstracts, replies, or corrections), those lacking adequate data, review articles, or studies of low methodological quality were excluded from the final selection.

### 2.3 Data abstraction

To ensure accuracy, data were independently collected by two authors. Any discrepancies were addressed through consultation with a third author. For each included study, the following information was retrieved: the first author’s name, publication year, country, study design, population’s characteristics, sample size, age, sex, NLR cut-off value, and ORs with 95% CIs from multivariate analyses. If any data were missing or incomplete, the corresponding authors were approached to for the full dataset, if accessible.

### 2.4 Quality evaluation

An evaluation of methodological quality was conducted through the application of the Newcastle-Ottawa Scale (NOS) (17). A quality score ranging from 7 to 9 on the NOS indicated high quality (18), whereas entries with scores below 7 were excluded from the meta-analysis. Two reviewers independently conducted the quality assessment, and discrepancies were resolved after consultation among the authors.

### 2.5 Statistical analysis

The meta-analysis was performed using Review Manager 5.4.1. OR with 95% CIs were applied to synthesize the data. Heterogeneity across outcomes was assessed using the chi-squared (χ2) test (Cochran’s Q) and inconsistency index (I^2^) (19). High heterogeneity was defined as a χ2 *p* value less than 0.1 or an I^2^ value exceeding 50%. To calculate the overall OR and 95% CI, a random-effects model was applied. Additionally, sensitivity and subgroup analyses were conducted to assess the stability of the results and identify potential sources of heterogeneity. Subgroup analyses were performed based on prespecified hypotheses (including cutoff and sample size). After categorization, the Generic Inverse Variance method in Review Manager 5.4.1 software was used, again employing a random-effects model, to calculate the pooled OR and 95% CI for each subgroup. Heterogeneity within subgroups was assessed using the Cochran’s Q test and the I^2^ statistic. We assessed publication bias by creating funnel plots in Review Manager 5.4.1 and conducting Egger’s regression tests (17) through Stata 15.1 for outcomes with 3 or more articles included. For publication bias, a *p* value under 0.05 was regarded as statistically significant.

## 3 Results

### 3.1 Literature retrieval and study characteristics

**Figure 1:**
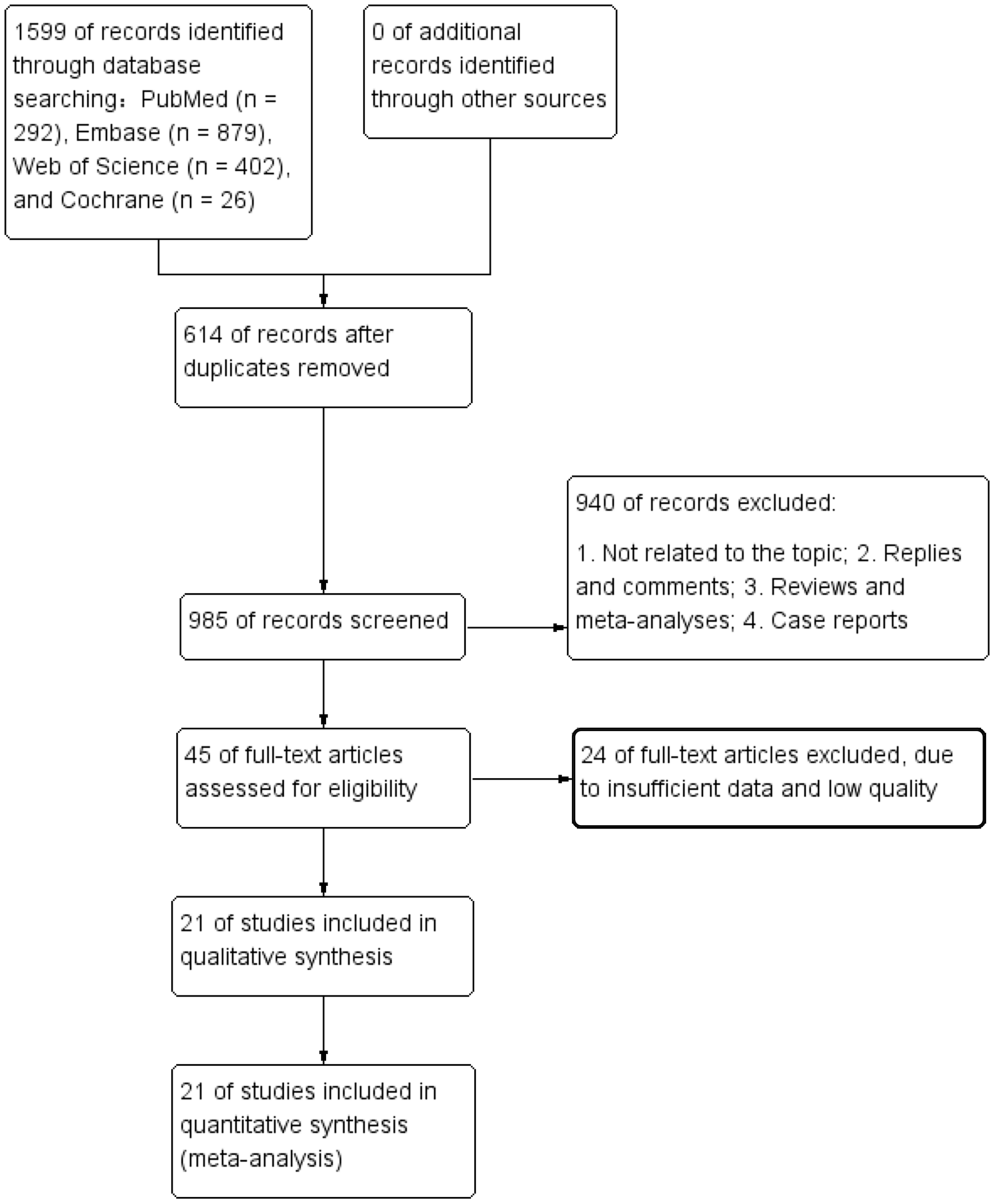
Flowchart of the systematic search and selection process.

The flowchart in Figure 1 outlines the process of literature retrieval and selection. At the beginning 1,599 studies were identified through systematic searches in PubMed (*n* = 292), Embase (*n* = 879), Web of Science (*n* = 402), Cochrane (*n* = 26). After removing duplicates, 985 titles and abstracts were analysed. Finally, 21 studies involving 23,621 patients were incorporated into our meta-analysis (10, 15, 18, 20–28). The characteristics and quality assessments of all research articles are summarized in Table 1.

**Table 1:** Characteristics and quality assessment of included studies.

Study	Region	Study design	Population	No. of patients	Gender		Mean/median age	NLR cut-off	NOS score
					Male	Female			
Akatsuka et al. (2021) (50)	Japan	Retrospective	Adult patients with sepsis and septic shock	238	158	80	67	NA	8
Bou Chebl et al. (2025) (10)	USA	Prospective	Adult patients with sepsis	874	515	359	73.4	14.15	8
He et al. (2025) (15)	China	Retrospective	Adult patients with sepsis	377	186	191	68	15.1	7
Hwang et al. (2017) (20)	Korea	Retrospective	Critically-ill adult septic patients	1,395	787	608	65	NA	7
Jin et al. (2024) (21)	China	Retrospective	Elderly patients with severe sepsis combined with diabetes mellitus	162	95	67	70.81	3.482	8
Li et al. (2024) (22)	China	Retrospective	Adult septic patients with coronary artery disease	1,175	749	426	71.46	12.58	7
Liang et al. (2022) (23)	China	Retrospective	Adult patients with bloodstream infections and sepsis	146	86	60	63.32	0.476	7
Liu et al. (2021) (24)	China	Retrospective	Adult patients with sepsis caused by intra-abdominal infection	216	116	100	54.7	4.18	7
Liu et al. (2021) (25)	China	Retrospective	Adult patients with sepsis	264	167	97	52.94	5.55	7
Liu et al. (2016) (26)	China	Prospective	Adult patients with sepsis	333	188	145	70.26	23.8	7
Mangalesh et al. (2023) (27)	India	Retrospective	Adult patients with sepsis	267	NA	NA	NA	NA	7
Qiu et al. (2024) (28)	China	Retrospective	Sepsis adult patients with lymphopenia	172	130	42	57.57	18.93	8
Ren (2022) (54)	China	Retrospective	ICU adult patients with sepsis and lung infection	1,676	1,007	669	58.85	NA	7
Sarı et al. (2019) (47)	Turkey	Retrospective	Septic shock adult patients in the intensive care unit	591	381	210	65	15	7
Shi et al. (2022) (37)	China	Retrospective	Adult patients with sepsis	173	123	50	64	15.85	7
Wen (2024) (55)	China	Retrospective	Adult patients with sepsis	606	375	231	56.67	14.395	7
Ye (2020) (56)	China	Retrospective	Adult patients with sepsis	3,043	1,539	1,504	67	20.25	7
Zhang (2024)-I (57)	China	Retrospective	Adult patients with sepsis	3,921	2,200	1721	60.9	NA	7
Zhang (2024)-II (58)	China	Retrospective	Sepsis patients in the intensive care unit	1,066	666	400	75	16.11	7
Zhang (2024)-III (59)	China	Retrospective	Adult patients with sepsis	1,263	732	531	66.23	NA	7
Zhao (2020) (60)	China	Retrospective	Septic patients in the emergency department	5,663	2,963	2,700	68	9.8	7

### 3.2 Meta-analysis

**Figure 2:**
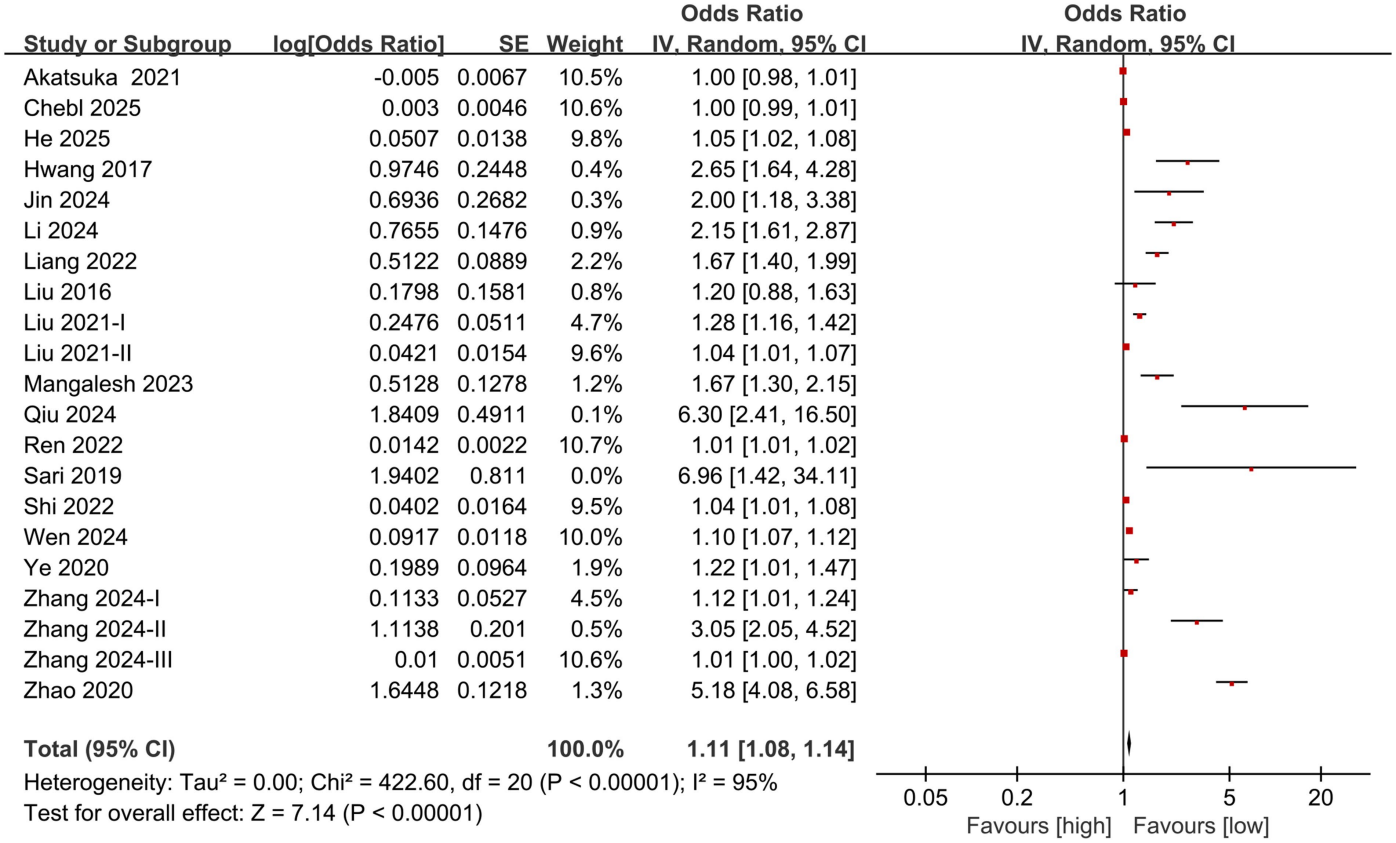
Forest plots of the association between NLR and mortality.

The division into high NLR group and low NLR group was based on the optimal cutoff value for prognosis prediction determined by receiver operating characteristic (ROC) curve analysis in each original study. For individuals diagnosed with sepsis, the meta-analysis of multivariate data revealed a significantly greater mortality rate in the high NLR group in comparison to the low NLR group (OR: 1.11; 95% CI: 1.08, 1.14; *p*<0.00001). A significant heterogeneity was identified (*I*^2^ = 95%, *p* <0.00001) (Figure 2).

### 3.3 Subgroup analysis

**Figure 3:**
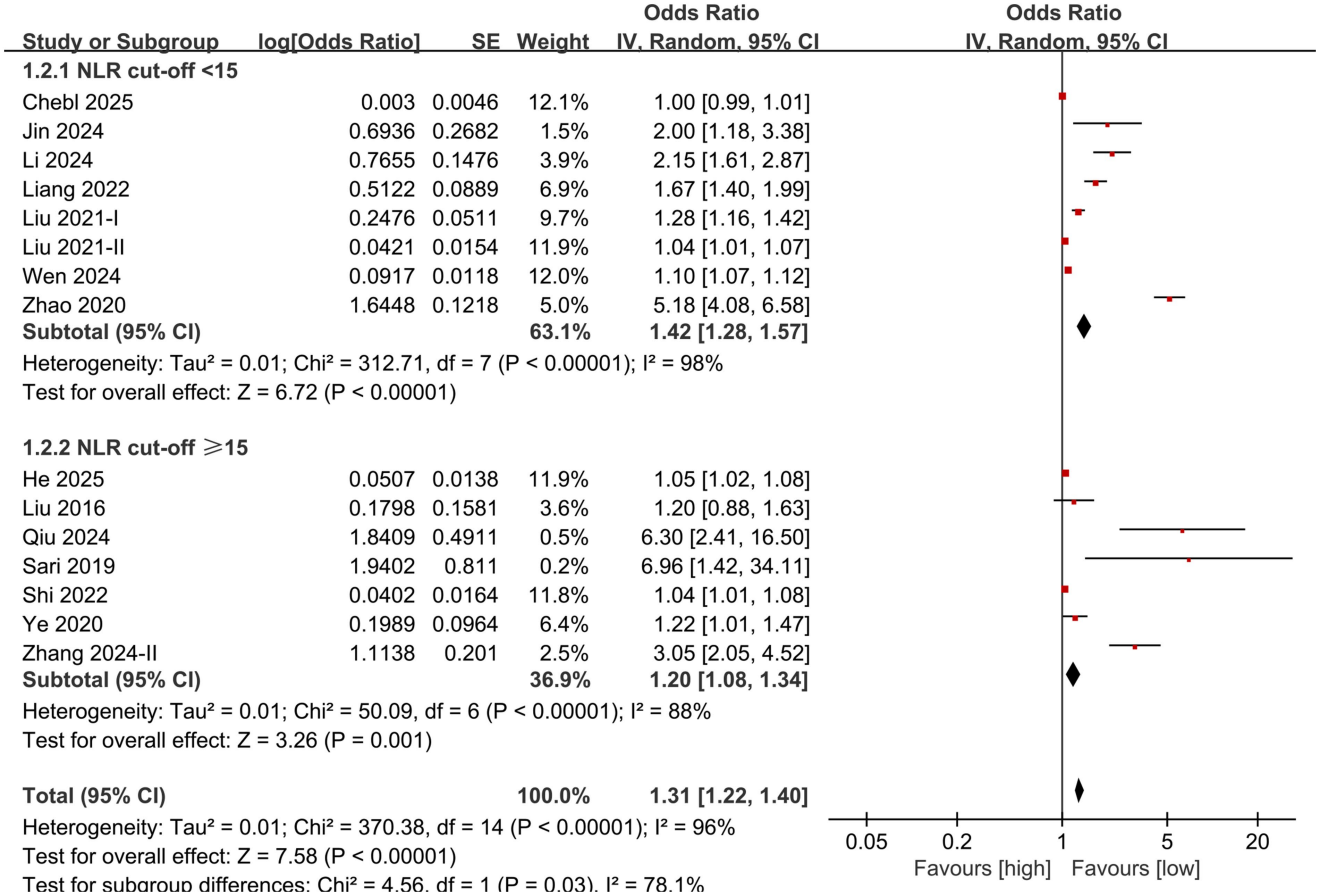
Forest plots of subgroup analysis based on NLR cut-off.

**Figure 4:**
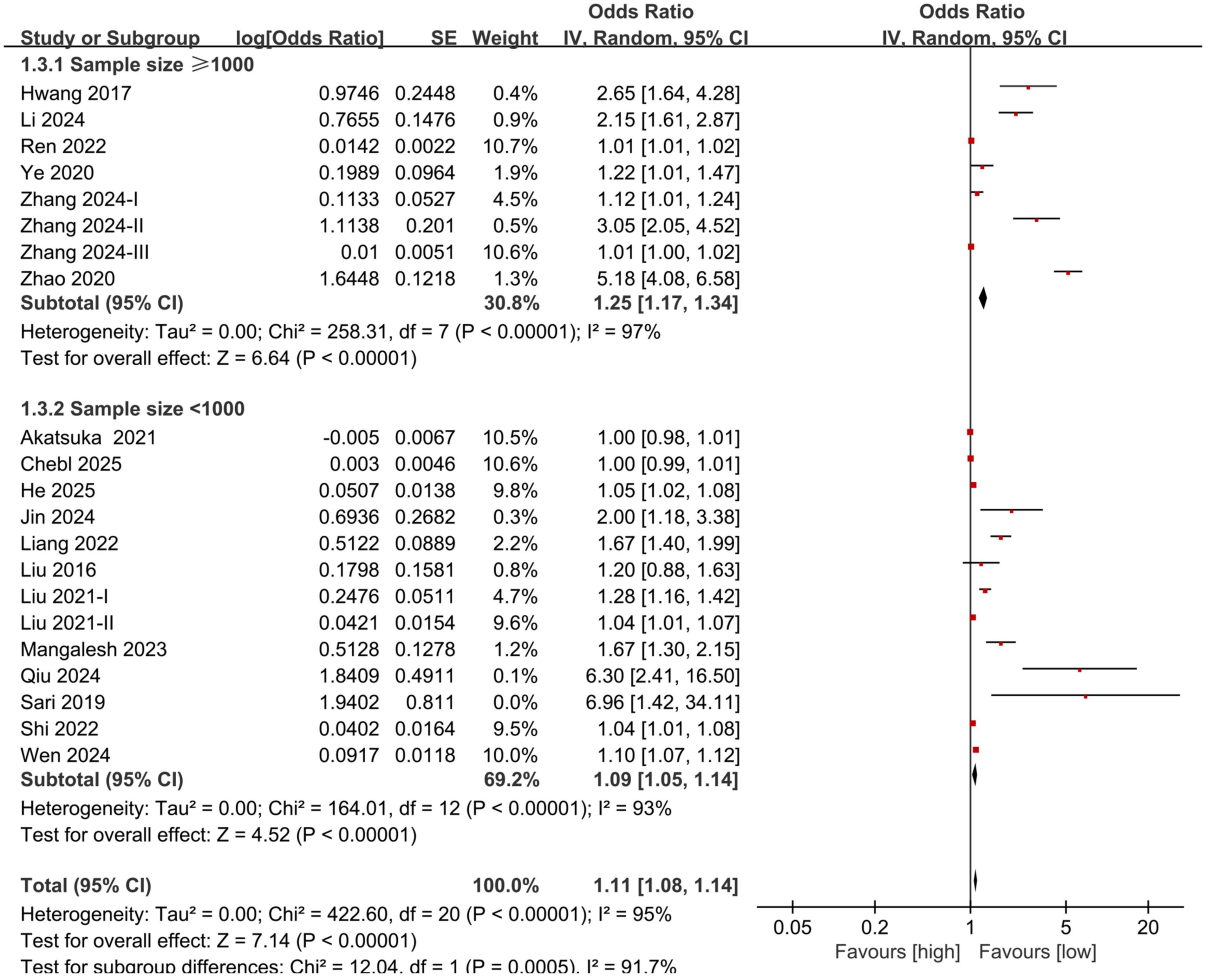
Forest plots of subgroup analysis based on sample size.

Subgroup analysis based on NLR cut-off and sample size was performed. The results showed that the predictive value of NLR for mortality in individuals diagnosed with sepsis was statistically significant in the subgroups with cut-off <15 (OR: 1.42; 95% CI: 1.28, 1.57), cut-off ≥15 (OR: 1.20; 95% CI: 1.08, 1.34) (Figure 3), sample size <1,000 (OR: 1.09; 95% CI: 1.05, 1.14), and sample size ≥1,000 (OR: 1.25; 95% CI: 1.17, 1.34) (Figure 4).

### 3.4 Publication bias and sensitivity analysis

**Figure 5:**
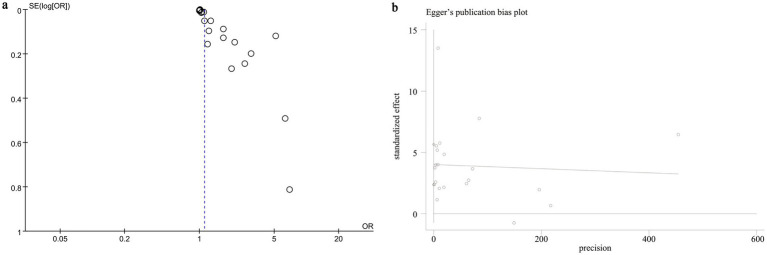
**(a)** Funnel plots and **(b)** Egger’s test plot.

**Figure 6:**
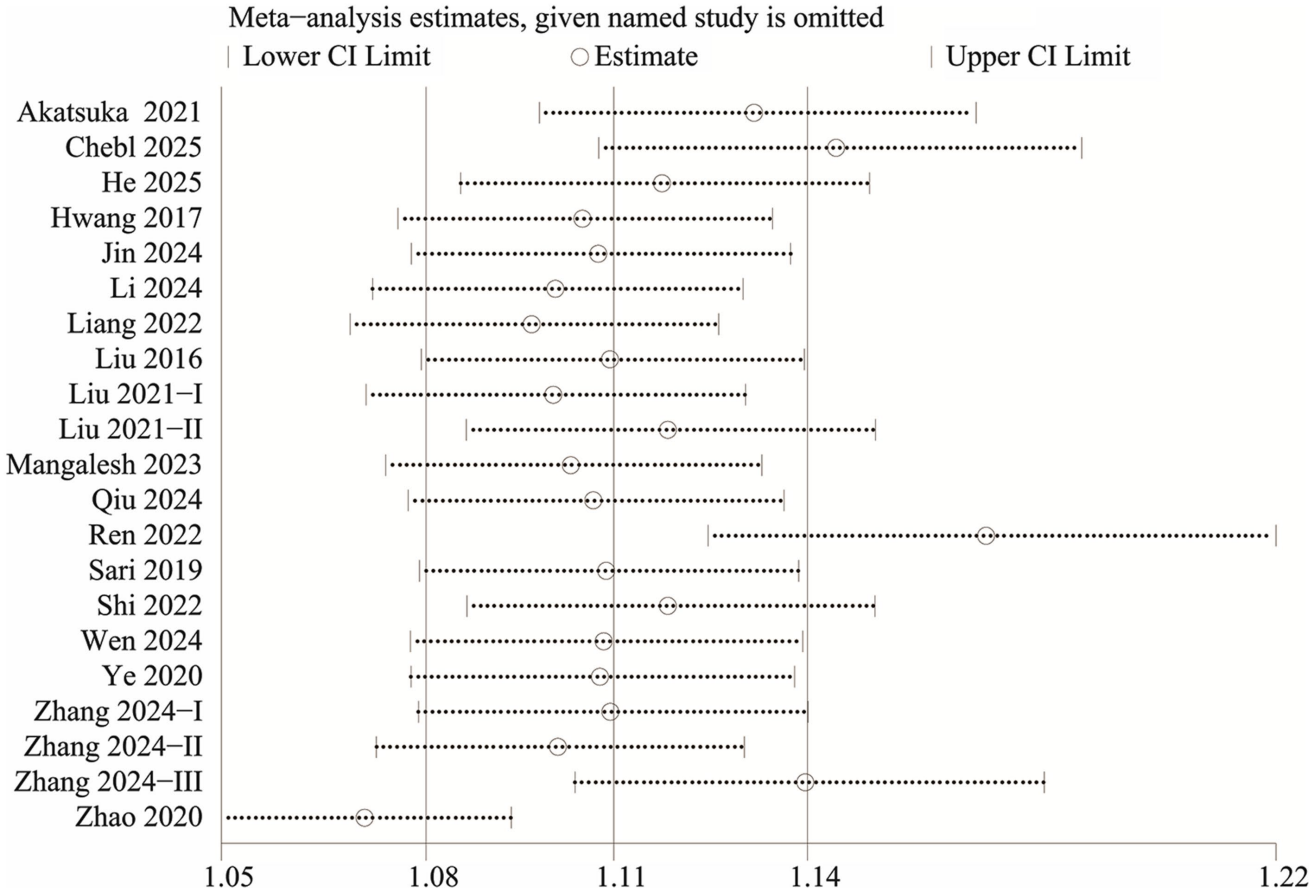
Sensitivity analysis.

We evaluated potential publication bias using funnel plots and Egger’s regression analysis. Both the funnel plot (Figure 5a) and the Egger’s test (*p* < 0.0001, Figure 5b) indicated significant publication bias for the relationship between NLR and mortality. Sensitivity analysis was performed by sequentially excluding each included study to examine their individual impact on the overall OR. The results demonstrated that the pooled OR remained consistent, suggesting that no single study significantly influenced the overall estimate (Figure 6).

## 4 Discussion

A routine blood test is one of the most common and simplest laboratory tests, providing valuable information about blood cells that can be read from the blood smear (29). The blood cell subtype ratios derived from routine blood test parameters, such as NLR, are valuable indicators (30, 31). Furthermore, because the NLR is the ratio of two absolute cell counts, any physiological or pathological condition that affects neutrophils or lymphocytes will systemically alter the NLR. Such conditions include, but are not limited to, urgent myelopoiesis and rapid mobilization of neutrophils from the bone marrow during acute inflammatory responses, as well as lymphocyte redistribution or apoptosis (32, 33). Currently, NLR has been used in the prognosis of individuals diagnosed with sepsis, but there is no consensus on its accuracy and clinical usefulness (13, 34). Therefore, the aim of this analysis is to evaluate the predictive value of NLR for mortality in individuals with sepsis through a systematic review and meta-analysis, and to deliver the most up-to-date and complete evidence-based basis for constructing an accurate prognostic model for individuals with sepsis.

This meta-analysis of 21 studies revealed that the mortality rate of sepsis patients with elevated NLR was significantly higher compared to those with lower NLR values, with sensitivity analysis confirming the stability of this result. Subgroup analysis by cut-off and sample size showed that the predictive value of NLR for mortality stayed significant in all subgroups. The subgroup analysis based on cut-off values indicated a decrease in heterogeneity, suggesting that the inconsistency of cut-off values may be one of the sources of high heterogeneity, but not the only one. In addition, sepsis is extremely heterogeneous. Factors such as its cause (such as infection site), type of pathogen (such as bacteria, fungi, viruses), patient’s underlying disease, and treatment regimen may significantly affect the baseline level and prognostic value of NLR, which may be an important reason for the high heterogeneity observed in this study. The remaining heterogeneity may be attributed to factors such as age, race, inclusion criteria, course of disease, and severity of sepsis, which needs to be verified by further large-scale studies. The conclusions of this study are consistent with those of Wu et al. (16). Therefore, based on the existing evidence, it can be inferred that NLR is indeed a predictive marker for the risk of death in individuals diagnosed with sepsis. In the clinical management of patients with sepsis, attention should be paid to the initial level and changes in NLR, in order to identify patients with poor prognosis early and take proactive measures.

Neutrophils and lymphocytes are the core of innate immunity and adaptive immunity, respectively. During sepsis infection, neutrophils reflect the inflammatory state, lymphocytes reflect immune function, and NLR captures the dynamic balance between inflammation and immunity (35), thereby reflecting the interplay between the body’s inflammatory response and immune state. Consistent with the present meta-analysis, prior research have shown that NLR is a prognostic marker for mortality in sepsis, including hospital mortality, 28-day mortality, 30-day mortality, and 90-day mortality (36, 37). In addition, Li et al. found that the combination of NLR and monocytes to high-density lipoprotein had a larger AUC than a single variable in predicting 28-day mortality in sepsis patients, with improved sensitivity and specificity (38). Lin et al. also found that NLR combined with RDW had a larger AUC for predicting death in emergency sepsis patients, though the sensitivity and specificity were not optimal (39). Currently, the optimal critical value of NLR for predicting mortality in individuals with sepsis ranges from 4.36 to 23.8 (40). However, a clear critical value needs further validation before it can be widely used in clinical practice. Additionally, some research has also found that NLR may serve as a biomarker for the severity, and treatment response of sepsis. In terms of severity assessment, Hou et al. and Martins et al. demonstrated that NLR can be used as an indicator for early identification of sepsis in the emergency department and ICU (41, 42). Furthermore, Meshaa et al. and Kriplani et al. found that NLR is an early predictor for identifying sepsis, regardless of its infectious source (43, 44).

In terms of sepsis severity, some research has shown that NLR is associated with the severity of sepsis as assessed by the APACHE II score, SOFA score, Simplified Acute Physiology II (SAPS II), and soluble leukocyte differentiation antigen 14 subtype (45, 46). Regarding sepsis treatment, Sari et al. observed that, after empirical antibiotic treatment, the NLR of patients with sepsis or septic shock who did not respond to treatment was significantly increased on the third day. They suggested using NLR in the first three days to evaluate and monitor the effect of antibiotic treatment in sepsis patients (47). In addition, NLR’s predictive and prognostic ability extends beyond adults. Recent clinical studies focusing on neonatal sepsis have demonstrated that NLR also demonstrates significant value in diagnosing neonatal sepsis (48–50). For example, Li et al. demonstrated that elevated NLR is associated with an increased risk of neonatal sepsis (51). Unfortunately, no data on the relationship between NLR and mortality in neonatal or pediatric sepsis were found in this study’s literature screening process. Therefore, it remains unclear whether NLR’s predictive value for mortality can be applied to neonates or children, requiring further research to clarify. Furthermore, because NLR is the ratio between two absolute cell counts, any physiological condition that affects neutrophils or lymphocytes will systemically alter the NLR. Acute granulopoiesis is a function of general severe inflammation, and changes in NLR can be observed in severe inflammatory response syndromes including acute pancreatitis and out-of-hospital cardiac arrest. In addition, conditions such as hematological malignancies, immunodeficiency, and the use of immunomodulatory drugs may also lead to changes in NLR (52). In recent years, advances in the study of sepsis endotypes have provided insights into the heterogeneity of its clinical and immune phenotypes. Patients with different endotypes may exhibit distinct immune responses, which may be one of the fundamental reasons for the heterogeneity in the prognostic value of inflammatory markers such as NLR (53). In clinical practice, careful interpretation of each test result is essential, considering the various factors that may affect NLR results.

## 5 Limitations

This study has revealed, to some extent, the predictive value of NLR as a frequently applied clinical hematological index for the ten-year mortality rate of patients with sepsis, but there are still some limitations. First, there is no unified standard for the selection and calculation of the optimal cut-off of NLR. The ROC curve method and the median method are commonly used methods in statistics. Heterogeneity in the findings may be attributed to the diverse approaches used to calculate NLR cut-off values and the wide range of case numbers reported across studies. While this analysis included a subgroup analysis based on the cut-off, it did not fully explain all the heterogeneity. Secondly, all participants in this meta-analysis were diagnosed with sepsis, the basic characteristics, etiology, and treatment methods of the patients included in each literature were different, which may lead to inevitable heterogeneity. The majority of the studies included in this study were single-center studies in Asia, lacking representative data from other regions, such as North America and Europe. This limits the global applicability of the study’s conclusions. This geographical imbalance may reflect differences in research focus or potential publication bias across regions, an important factor to consider when interpreting the results of this study. Even with these limitations, our analysis is currently the most up-to-date and the largest evidence-based analysis on the relationship between NLR and the risk of death in individuals diagnosed with sepsis. Our analysis emphasizes the need to monitor changes in NLR levels in the clinical treatment of individuals diagnosed with sepsis and to establish a more effective prediction model that incorporates NLR to maximize the prognosis of patients with sepsis and reduce the risk of death.

## 6 Conclusion

The meta-analysis results highlighted a significant association between NLR and the risk of death in patients with sepsis, with higher NLR values corresponding to an increased mortality risk. Given the potential for publication bias and the unavoidable heterogeneity observed in this study, further large-scale, multicenter, prospective clinical studies are needed to confirm the relationship between NLR and mortality risk in sepsis patients. Additionally, the prognostic value of NLR in neonatal and pediatric sepsis remains an area requiring further investigation.

## Data Availability

The original contributions presented in the study are included in the article/Supplementary material, further inquiries can be directed to the corresponding author.
