# Supplementary material for: Association between neutrophil to lymphocyte ratio and the mortality of patients with sepsis: an update systematic review and meta-analysis
Source: Front Med (Lausanne). 2025 Oct 20;12:1637365. doi: 10.3389/fmed.2025.1637365 (PMC12580286; doi:10.3389/fmed.2025.1637365)
Supplement: Supplementary file 1 [file Data_Sheet_1.DOCX]

TableS1 Literature search strategy

Pubmed-292

((((("Neutrophils"[Mesh]) OR (((Neutrophil) OR (Polymorphonuclear Leukocyte)) OR (LE Cell))) AND (("Lymphocytes"[Mesh]) OR ((Lymphocyte) OR (Lymphoid Cell)))) AND (Ratio)) AND (("Sepsis"[Mesh]) OR ((((((Bloodstream Infection) OR (Septicemia)) OR (Blood Poisoning)) OR (Pyemia)) OR (Pyaemia)) OR (Pyohemia)))) AND (("Mortality"[Mesh]) OR (((Mortalities) OR (Death Rate)) OR (Death)))

Embase-879

((Neutrophils or (Neutrophil or Polymorphonuclear Leukocyte or LE Cell)) and (Lymphocytes or (Lymphocyte or Lymphoid Cell)) and Ratio and (Sepsis or (Bloodstream Infection or Septicemia or Blood Poisoning or Pyemia or Pyaemia or Pyohemia)) and (Mortality or (Mortalities or Death Rate or Death))).af.

Cochrane-26

((Neutrophils or (Neutrophil or Polymorphonuclear Leukocyte or LE Cell)) and (Lymphocytes or (Lymphocyte or Lymphoid Cell)) and Ratio and (Sepsis or (Bloodstream Infection or Septicemia or Blood Poisoning or Pyemia or Pyaemia or Pyohemia)) and (Mortality or (Mortalities or Death Rate or Death))).af.

Web of Science-402

(((((Neutrophils) OR (((Neutrophil) OR (Polymorphonuclear Leukocyte)) OR (LE Cell))) AND ((Lymphocytes) OR ((Lymphocyte) OR (Lymphoid Cell)))) AND (Ratio)) AND ((Sepsis) OR ((((((Bloodstream Infection) OR (Septicemia)) OR (Blood Poisoning)) OR (Pyemia)) OR (Pyaemia)) OR (Pyohemia)))) AND ((Mortality) OR (((Mortalities) OR (Death Rate)) OR (Death))) (Topic)
